# Evaluation of Adjuvant Treatments for T1 N0 M0 Triple-Negative Breast Cancer

**DOI:** 10.1001/jamanetworkopen.2020.21881

**Published:** 2020-11-19

**Authors:** Zhen Zhai, Yi Zheng, Jia Yao, Yu Liu, Jian Ruan, Yujiao Deng, Linghui Zhou, Peng Zhao, Si Yang, Jingjing Hu, Bajin We, Ying Wu, Dai Zhang, Huafeng Kang, Zhijun Dai

**Affiliations:** 1 Department of Breast Surgery, The First Affiliated Hospital, College of Medicine, Zhejiang University, Hangzhou, China; 2 Department of Oncology, The Second Affiliated Hospital of Xi’an Jiaotong University, Xi’an, China; 3 Department of Medical Oncology, The First Affiliated Hospital, College of Medicine, Zhejiang University, Hangzhou, China; 4 Dana-Farber Cancer Institute, Harvard Medical School, Boston, Massachusetts

## Abstract

**Importance:**

Adjuvant chemotherapy remains the only recommended treatment for patients with triple-negative breast cancer (TNBC). However, the existing evidence is not enough to recommend adjuvant therapies to patients with T1 N0 M0 TNBC.

**Objective:**

To evaluate the association of different adjuvant therapies with survival outcome in patients with T1 N0 M0 TNBC stratified by cancer stage and age.

**Design, Setting, and Participants:**

Postoperative patients diagnosed as having T1 N0 M0 TNBC between 2010 and 2015 who were enrolled in the Surveillance, Epidemiology, and End Results cancer registry program were included in this population-based cohort study. Data analysis was performed from March 27, 2019, to August 10, 2020.

**Exposures:**

Chemotherapy and radiotherapy.

**Main Outcomes and Measures:**

Kaplan-Meier curve and univariate and multivariable Cox proportional hazards regression analyses were performed to compare overall survival (OS) and breast cancer-specific survival (BCSS) between the different treatments.

**Results:**

A cohort of 7739 eligible patients (mean [SD] age, 59.5 [12.4] years; all female) were included in the present study. The 5-year OS of the total patients was 91.7% (95% CI, 90.9%-92.5%), and median follow-up was 45 months (95% CI, 44-46 months). Patients aged 70 years and older or with T1a TNBC were more likely to receive adjuvant radiotherapy than chemotherapy. Although any adjuvant therapy could improve OS in T1 N0 M0 TNBC, only chemotherapy was associated with significantly better breast cancer–specific survival (BCSS adjusted hazard ratio: 0.657; 95% CI, 0.460-0.939; *P* = .02). Adjuvant radiotherapy after breast-conserving surgery was associated with better OS and BCSS in patients aged 70 years and older but not in those younger than 70 years. For patients with T1c BC, chemotherapy after breast-conserving surgery or other surgery was associated with improved OS, whereas only chemotherapy after other surgery was associated with better BCSS.

**Conclusions and Relevance:**

The findings of this cohort study suggest that adjuvant therapies could improve OS in patients with T1 N0 M0 TNBC, whereas only chemotherapy was associated with better BCSS. Older patients with early-stage TNBC may benefit from adjuvant radiotherapy. Administration of adjuvant therapies to patients with different ages and cancer stages should be discussed carefully, which necessitates guidance from updated guidelines.

## Key Points

**Question:**

Should patients with T1 N0 M0 triple-negative breast cancer (TNBC) receive routine adjuvant chemotherapy and radiotherapy after surgery?

**Findings:**

In this cohort study of 7739 postoperative patients diagnosed as having T1 N0 M0 TNBC from the Surveillance, Epidemiology, and End Results cancer registry program, receipt of adjuvant therapies was associated with an overall survival benefit. Adjuvant radiotherapy after breast-conserving surgery was associated with better overall and breast cancer–specific survival in patients aged 70 years and older but not in those younger than 70 years.

**Meaning:**

Administration of adjuvant therapies to patients with different ages and cancer stages should be discussed carefully, which necessitates guidance from updated guidelines.

## Teaser

This cohort study evaluates the association of different adjuvant therapies with survival outcomes in patients with triple-negative breast cancer.

## Introduction

Breast cancer (BC) is the most commonly diagnosed cancer in women and the leading cause of cancer-related deaths in women in most countries.^1^ The increase in BC incidence was largely attributable to the change in reproductive patterns, such as fewer births and delayed childbearing, and partly due to the increased use of mammography screening.^2,3^ The detection rate of breast tumors less than 2 cm in size has increased greatly with the advent of mammography screening. However, most small tumors do not grow in size and lead to clinical symptoms.^4^ A multi-institutional study reported favorable prognosis in patients with small breast tumors despite not receiving chemotherapy.^5^ Despite the benign prognosis in patients with early-stage BC, the recurrence risk varied when tumors were divided into subgroups using hormone receptor and human epidermal growth factor receptor 2 (ERBB2 [formerly HER2]). Compared with other subtypes, patients with triple-negative BC (TNBC), which accounts for nearly 20% of all BC cases, were expected to have poorer survival outcome and higher recurrence risk.^5,6,7^ Thus, whether patients with T1 N0 M0 TNBC require routine adjuvant chemotherapy and radiotherapy after surgery remains unclear.

Previous studies revealed that for patients with T1 N0 M0 TNBC, receiving no systemic therapies was associated with a substantial recurrence risk, and delayed initiation of adjuvant chemotherapy was associated with adverse outcomes.^8,9^ For these patients, the National Comprehensive Cancer Network (NCCN) guidelines for BC (Version 4.2020)^10^ recommend adjuvant chemotherapy for tumors larger than 1 cm in size, indicating the role of tumor stage in treatment decisions. However, radiotherapy was not recommended for patients with early-stage TNBC, whereas a number of studies reported improvements of local control and long-term survival in patients with TNBC with postoperative radiation therapy.^11^ Thus, the benefit of adjuvant radiotherapy in patients with T1 N0 M0 TNBC remains controversial and requires further investigation.^12^ In addition, adjuvant therapies, including chemotherapy, were not considered the standard treatment for patients aged 70 years and older in NCCN guidelines.^10^ A historical cohort study^13^ reported survival benefit of adjuvant radiotherapy in patients younger than 40 years with TNBC, but not in older patients. However, another population-based study^14^ reported improvements in BC-specific survival (BCSS) for patients aged 70 years and older after adjuvant radiotherapy. Taken together, the present study was aimed at evaluating the association of adjuvant therapies, including adjuvant chemotherapy and radiotherapy, with overall survival (OS) and BCSS in patients with T1 N0 M0 TNBC stratified by cancer stage and patient age.

## Methods

To evaluate the association of different adjuvant therapies after surgery with the survival of patients with T1 N0 M0 TNBC, data of eligible patients were extracted from Surveillance, Epidemiology, and End Results (SEER) 18 Regs Custom Data (with additional treatment fields) by using the SEER*Stat 8.3.6 software. As the largest publicly available cancer data set, the SEER collected information from 18 population-based cancer registries from 1975 to 2016 and provided deidentified information on cancer statistics of the US population. Patients in the SEER were followed until death, and any patient that dies after the follow-up cutoff date is recoded to alive as of the cutoff date. Patient staging was verified as per the seventh edition of American Joint Committee on Cancer (AJCC) staging system 2010.^15^ Tumors with the largest diameters reported as 2 cm or smaller in the gross pathological report were defined as stage T1 and were classified as T1a (1-5 mm), T1b (6-10 mm), and T1c (11-20 mm), whereas T1mic and Tis were excluded. Stage N0 and M0 were defined as no regional lymph node metastases and distant metastases, respectively. We only included patients who underwent axillary node dissection to confirm their node-negative status. TNBC was defined as estrogen receptor (ER) negative, progesterone receptor (PR) negative, and ERBB2 negative. ER and PR were considered negative if less than 1% of cells stained positive. ERBB2 status was considered negative if immunohistochemistry score was 0 to 1+ or there was no amplification on fluorescence in situ hybridization or chromogenic in situ hybridization. Patients with missing hormone receptor status or ERBB2 status and those with borderline or equivocal results were excluded.

After excluding patients diagnosed at autopsy or in death certificates only, data of 11 395 patients aged 18 years and older who had T1 N0 M0 TNBC were extracted between 2010 and 2015. We selected patients with 1 primary malignant neoplasm only and retrieved individual characteristics, including age, sex, race/ethnicity, marital status, tumor grade, AJCC Seventh T stage, surgery, chemotherapy, and radiotherapy. Race/ethnicity as defined by the SEER was included to make the results more generalizable to the US population. Patients with unknown characteristics were excluded, as well as male patients, as there were only 6 of them. After the patient selection, 7739 eligible patients were included. We integrated the chemotherapy and radiotherapy data as adjuvant therapy, as many patients received both therapies. The Ethics Committee of the First Affiliated Hospital, College of Medicine, Zhejiang University approved this study. The access to and use of SEER data did not require informed patient consent. This study followed the Strengthening the Reporting of Observational Studies in Epidemiology (STROBE) reporting guideline for cohort studies.

Frequencies and proportions were calculated to describe the baseline characteristics of eligible patients, and categorical variables were compared between the different stages using the χ^2^ test. The 5-year OS and BCSS of the total patients were investigated. To investigate the influence of adjuvant therapies on patient prognosis, Kaplan-Meier survival curves and cumulative hazard curves were generated, with further log-rank tests. In the further univariate and multivariable Cox proportional analyses, hazard ratios (HR) and their corresponding 95% CIs were calculated to evaluate the association of adjuvant therapies with OS and BCSS. To verify the NCCN guidelines for TNBC, we then calculated the adjusted hazard ratios (AHR) and corresponding 95% CIs for patients who received adjuvant therapies compared with those who did not, stratified by age and cancer stage, to handle potential biases and evaluate the prognostic association of adjuvant therapies. Data analysis was performed from March 27, 2019, to August 10, 2020. A 2-sided *P* value of <.05 indicated a significant difference. All calculations were performed, and figures were generated, using R version 3.5.3 software (R Project for Statistical Computing).

## Results

### Patients Baseline Characteristics

**Figure 1.**
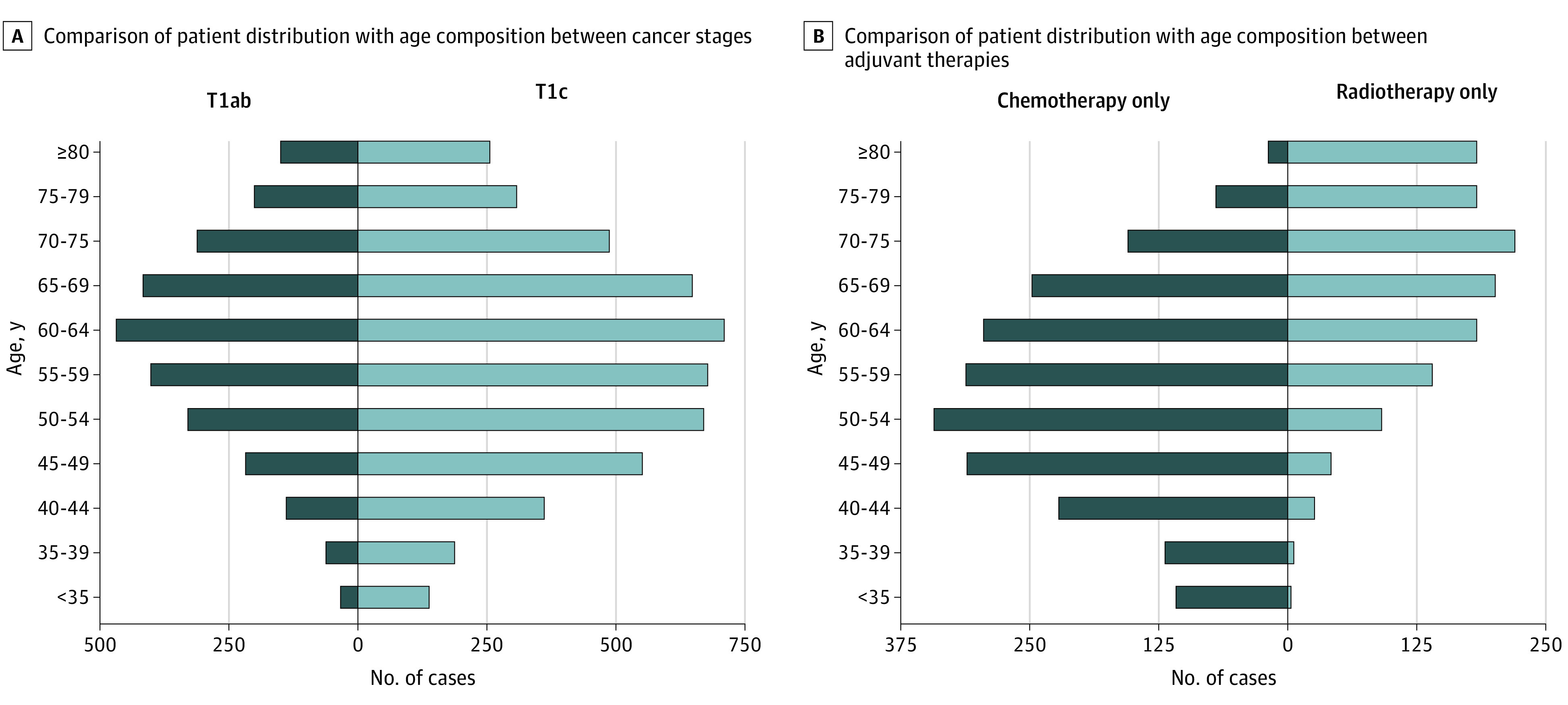
Comparison of Patient Distribution With Age Composition Between Different Cancer Stages and Adjuvant Therapies

In view of the inclusion criteria, the present study included 7739 patients (mean [SD] age, 59.5 [12.4] years; all female) diagnosed as having T1 N0 M0 TNBC between 2010 and 2015 who underwent BC surgery. A detailed flowchart of the patient selection process is presented in eFigure in the Supplement. The 5-year OS and BCSS of the total patients were 91.7% (95% CI, 90.9%-92.5%) and 94.9% (95% CI, 94.3%-95.6%), respectively, with 461 deaths recorded, of which 270 were attributable to BC. Significant distribution differences in age, race/ethnicity, grade, surgery, adjuvant therapy, vital status, and cause of death were observed between the T1a, T1b, and T1c subgroups (Table 1). Among the eligible patients, the cancer stage was T1a in 755 (9.8%), T1b in 1979 (25.6%), and T1c in 5005 (64.7%). As shown in Table 1, most enrolled patients were aged 50 to 70 years, had White ethnicity, were married, had grade 3 tumors; and received breast-conserving surgery (BCS). More patients had grade 3 tumors in the T1c subgroup than in the T1a and T1b subgroups, whereas a higher proportion of grade 2 tumors was found in the T1a subgroup. Patients with T1a TNBC were more likely to receive only radiotherapy (43.6%) after surgery, followed by no adjuvant therapy (31.4%). By contrast, patients with T1b and T1c BC mostly received both adjuvant therapies, whereas a large proportion (33.1%) of patients with T1c were treated with chemotherapy only. As expected, a higher proportion of deaths were attributable to BC advanced stages. As shown in Figure 1, most cases occurred in patients aged 60 to 64 years, and the number of patients was nearly normally distributed in terms of age. We observed that older patients preferred radiotherapy to chemotherapy, whereas patients younger than 70 years mostly chose chemotherapy over radiotherapy.

**Table 1.** Baseline Demographic Characteristics of Patients With T1 N0 M0 Triple-Negative Breast Cancer

Characteristic	Total	Stage			*P* value
		T1a	T1b	T1c	
Overall No.	7739	755	1979	5005	
Age, y					
<35	173	9 (1.2)	25 (1.3)	139 (2.8)	.002
35-50	1523	116 (15.4)	303 (15.3)	1104 (22.1)	
50-70	4328	439 (58.1)	1179 (59.6)	2710 (54.1)	
≥70	1715	191 (25.3)	472 (23.9)	1052 (21)	
Race/ethnicity					
Non-Hispanic White	4997	505 (66.9)	1332 (67.3)	3160 (63.1)	<.001
Non-Hispanic Black	1377	113 (15)	346 (17.5)	918 (18.3)	
Non-Hispanic other races	569	65 (8.6)	131 (6.6)	373 (7.5)	
Hispanic (all races)	796	72 (9.5)	170 (8.6)	554 (11.1)	
Marital status					
Married	4790	476 (63)	1251 (63.2)	3063 (61.2)	.33
Single	1064	104 (13.8)	248 (12.5)	712 (14.2)	
Divorced, separated, or widowed	1885	175 (23.2)	480 (24.3)	1230 (24.6)	
Grade					
G1	276	59 (7.8)	99 (5)	118 (2.4)	<.001
G2	1775	287 (38)	559 (28.2)	929 (18.6)	
G3	5644	405 (53.6)	1317 (66.5)	3922 (78.4)	
G4	44	4 (0.5)	4 (0.2)	36 (0.7)	
Surgery					
Breast conserving	5372	497 (65.8)	1453 (73.4)	3422 (68.4)	<.001
Radical	463	56 (7.4)	96 (4.9)	311 (6.2)	
Simple	1798	191 (25.3)	407 (20.6)	1200 (24)	
Other	106	11 (1.5)	23 (1.2)	72 (1.4)	
Adjuvant therapy					
None	1286	237 (31.4)	316 (16)	733 (14.6)	<.001
Chemotherapy	2202	82 (10.9)	462 (23.3)	1658 (33.1)	
Radiotherapy	1278	329 (43.6)	459 (23.2)	490 (9.8)	
Both	2973	107 (14.2)	742 (37.5)	2124 (42.4)	
Vital status					
Alive	7278	732 (97.0)	1894 (95.7)	4652 (92.9)	<.001
Deceased	461	23 (3.0)	85 (4.3)	353 (7.1)	
Cause of death					
Non–breast cancer	7469	746 (98.8)	1932 (97.6)	4791 (95.7)	<.001
Breast cancer	270	9 (1.2)	47 (2.4)	214 (4.3)	

### OS Outcomes

**Figure 2.**
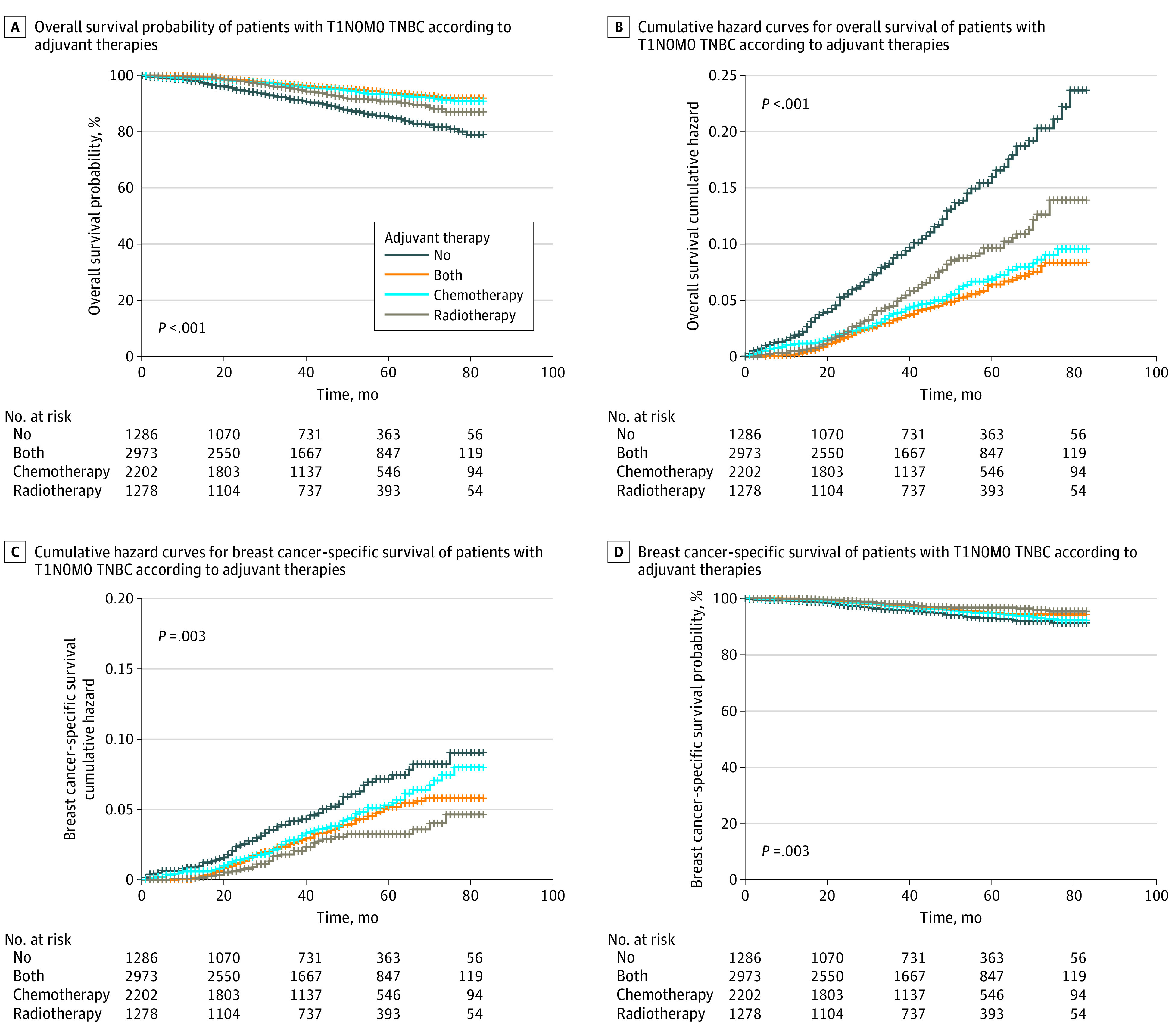
Kaplan-Meier Survival and Cumulative Hazard Curves The overall A, and breast cancer-specific survival B, of patients with T1 N0 M0 triple-negative breast cancer (TNBC) according to adjuvant therapies.

Kaplan-Meier and cumulative hazard curves were generated by adjuvant therapies for patients with T1 N0 M0 TNBC. Median follow-up for OS was 45 months (95% CI, 44-46 months). As shown in Figure 2A, patients who received chemotherapy, with or without radiotherapy, showed better OS than the other patients. Meanwhile, patients who received radiotherapy were associated with better OS and less hazard than those who received no adjuvant therapy. The univariate Cox analyses revealed significant differences between the subgroups in age, marital status, grade, stage, surgery, and adjuvant therapy. Considering the potential bias caused by mutual association between characteristics, a further multivariable Cox analysis was conducted. Age, marital status, grade, stage, surgery, and adjuvant therapy were identified to be independent risk factors associated with OS, while no significant difference in OS was observed between patients of different race/ethnicities (eTable in the Supplement).

Compared with patients aged 50 to 70 years, those who were aged 70 years and older had poorer OS (AHR, 2.090; 95% CI, 1.677-2.604; *P* < .001). As for marital status, divorced, separated, or widowed patients were associated with poorer OS than married patients (AHR, 1.385; 95% CI, 1.122-1.708; *P* = .002), whereas no significant difference was found between single and married patients. As expected higher tumor grade was associated with worse prognosis. The patients with T1c BC had an AHR of 2.829 (95% CI, 1.834-4.363; *P* < .001) as compared with patients with T1a, whereas no significant difference was observed between patients with T1b and T1a BC (AHR, 1.549; 95% CI, 0.973-2.466; *P* = .06). In the univariate analysis, OS was worse in patients who received a radical mastectomy than in those with BCS (HR, 1.484; 95% CI, 1.069-2.059; *P* = .02), whereas no significant difference was found between them in the multivariable analysis (AHR, 1.206; 95% CI, 0.833-1.744; *P* = .32). In addition, compared with patients who did not receive adjuvant therapy, those who received chemotherapy (AHR, 0.475; 95% CI, 0.362-0.625; *P* < .001), radiotherapy (AHR, 0.725; 95% CI, 0.527-0.996; *P* = .04), or both (AHR, 0.489; 95% CI, 0.362-0.661; *P* < .001) were all associated with better OS (eTable in the Supplement).

### BC-Specific Survival Outcomes

As shown in the Kaplan-Meier and cumulative hazard curves for BCSS, adjuvant therapy was associated with better prognosis than no therapy after surgery. In addition, patients who received only radiotherapy had better BCSS than other groups, but no significant difference was found between chemotherapy and both therapies (Figure 2B). The median follow-up for BCSS was 44 months (95% CI, 43-45 months). As shown in the multivariable analyses in Table 2, patients aged 70 years and older had poorer BCSS than younger patients (AHR, 1.464; 95% CI, 1.074-1.996; *P* = .02). There was no significant difference found in BCSS between marital subgroups. Although race/ethnicity was not identified as an independent risk factor of OS, a significantly worse BCSS was observed in Non-Hispanic Black patients compared with Non-Hispanic White patients (AHR, 1.402; 95% CI, 1.037-1.895; *P* = .03). Similarly, inconsistent with the OS results, patients who received radical mastectomy showed significantly worse BCSS compared with patients who received BCS (AHR, 1.847; 95% CI, 1.146-2.977; *P* = .01), whereas receiving simple mastectomy was related to worse OS (AHR, 1.325; 95% CI, 1.009-1.739; *P* = .04) (eTable in the Supplement) and BCSS (AHR, 1.889; 95% CI, 1.303-2.738, *P* < .001) compared with receiving BCS. As expected, patients with higher-grade tumor had significant worse BCSS compared with grade 1 BC. As for stage, patients with T1c BC had poorer BCSS (AHR, 3.568; 95% CI, 1.803-7.063; *P* < .001) than those with T1a BC, whereas no significant difference was found between patients with T1b and T1a BC (AHR, 1.982; 95% CI, 0.965-4.071; *P* = .06). In the multivariable analysis, patients who received chemotherapy only were associated with better BCSS (AHR, 0.657; 95% CI, 0.460-0.939; *P* = .02) than those who did not receive adjuvant therapy, whereas radiotherapy or both adjuvant therapies were not associated with improved BCSS (Table 2).

**Table 2.** Univariate and Multivariable Cox Analysis of Breast Cancer–Specific Survival in Patients With T1 N0 M0 Triple-Negative Breast Cancer

Characteristic	Patients, No.	Events, No.	Rate of events/patient, No.	Univariate analysis		Multivariable analysis	
				BCSS HR (95% CI)	*P* value	BCSS AHR (95% CI)	*P* value
Age, y							
50-70	4328	133	3.07	1 [Reference]		1 [Reference]	
<35	173	9	5.20	1.705 (0.868-3.348)	.12	1.339 (0.672-2.670)	.41
35-50	1523	53	3.48	1.126 (0.819-1.548)	.47	0.964 (0.695-1.338)	.83
≥70	1715	75	4.37	1.474 (1.110-1.956)	.007	1.464 (1.074-1.996)	.02
Race/ethnicity							
Non-Hispanic White	4997	164	3.28	1 [Reference]		1 [Reference]	
Non-Hispanic Black	1377	61	4.43	1.396 (1.041-1.873)	.03	1.402 (1.037-1.895)	.03
Non-Hispanic other races	569	15	2.64	0.847 (0.499-1.437)	.54	0.841 (0.495-1.429)	.52
Hispanic (all races)	796	30	3.77	1.271 (0.861-1.876)	.23	1.226 (0.826-1.819)	.31
Marital status							
Married	4790	158	3.30	1 [Reference]		1 [Reference]	
Single	1064	38	3.57	1.082 (0.759-1.541)	.66	1.008 (0.702-1.446)	.97
Divorced, separated, or widowed	1885	74	3.93	1.170 (0.891-1.547)	.26	1.033 (0.773-1.381)	.83
Grade							
G1	276	2	0.72	1 [Reference]		1 [Reference]	
G2	1775	53	2.99	4.361 (1.063-17.89)	.04	4.044 (0.983-16.643)	.05
G3	5644	211	3.74	5.567 (1.383-22.40)	.02	4.813 (1.188-19.495)	.03
G4	44	4	9.09	12.171 (2.229-66.45)	.004	9.463 (1.720-52.046)	.01
Stage							
T1a	755	9	1.19	1 [Reference]		1 [Reference]	
T1b	1979	47	2.37	1.882 (0.923-3.841)	.08	1.982 (0.965-4.071)	.06
T1c	5005	214	4.28	3.551 (1.823-6.919)	<.001	3.568 (1.803-7.063)	<.001
Surgery							
Breast conserving	5372	154	2.87	1 [Reference]		1 [Reference]	
Simple	1798	86	4.78	1.718 (1.319-2.236)	<.001	1.889 (1.303-2.738)	<.001
Radical	463	27	5.83	1.795 (1.192-2.702)	.005	1.847 (1.146-2.977)	.01
Other	106	3	2.83	1.446 (0.461-4.537)	.53	1.826 (0.566-5.892)	.31
Adjuvant therapy							
None	1286	64	4.98	1 [Reference]		1 [Reference]	
Chemotherapy	2202	78	3.54	0.754 (0.542-1.050)	.09	0.657 (0.460-0.939)	.02
Radiotherapy	1278	31	2.43	0.467 (0.304-0.718)	<.001	0.805 (0.489-1.327)	.40
Both	2973	97	3.26	0.648 (0.473-0.889)	.007	0.895 (0.593-1.351)	.60

Abbreviations: AHR, adjusted hazard ratio; HR, hazard ratio.

### Subgroup Analysis Stratified by Age and Cancer Stage

To address potential biases and evaluate the prognostic association of adjuvant therapies in different age and stage subgroups, we calculated AHRs for each therapy after different surgical procedures. We divided patients in to 2 groups, including the BCS and Other group (simple mastectomy, radical mastectomy, or other surgical procedures). For patients younger than 70 years, receiving chemotherapy after BCS (AHR, 0.574; 95% CI, 0.400-0.824; *P* = .003) or other procedures (AHR, 0.610; 95% CI, 0.395-0.941; *P* = .03) were both associated with improved OS rather than BCSS, whereas no significant survival benefit was found in patients who received radiotherapy after BCS. Furthermore, receiving radiotherapy after other surgery (including simple and radical mastectomy) was associated with worse OS (AHR, 2.514; 95% CI, 1.408-4.490; *P* = .002) and BCSS (AHR, 3.149; 95% CI, 1.708-5.805; *P* < .001). For patients aged70 years and older who had TNBC, for whom adjuvant therapies were not definitely recommended by the NCCN guidelines, all adjuvant therapies were associated with improved OS except for radiotherapy after other type of surgery (AHR, 1.324; 95% CI, 0.406-4.320; *P* = .64). However, only radiotherapy after BCS was associated with better BCSS (AHR, 0.478; 95% CI, 0.251-0.913; *P* = .03) for these patients (Table 3).

**Table 3.** Adjusted Hazard Ratio for OS and BCSS Associated With Adjuvant Therapies After Different Surgical Procedures in Patients With Different Cancer Stages and Ages

Characteristic	BCS				Other^a^			
	Chemotherapy		Radiotherapy		Chemotherapy		Radiotherapy	
	AHR (95% CI)	*P* Value	AHR (95% CI)	*P* Value	AHR (95% CI)	*P* Value	AHR (95% CI)	*P* Value
Age								
<70								
OS	0.574 (0.400-0.824)	.003	0.977 (0.673-1.417)	.9	0.610 (0.395-0.941)	.03	2.514 (1.408-4.490)	.002
BCSS	0.812 (0.512-1.287)	.38	1.023 (0.651-1.608)	.92	0.835 (0.493-1.413)	.50	3.149 (1.708-5.805)	<.001
≥70								
OS	0.464 (0.305-0.704)	<.001	0.507 (0.349-0.736)	<.001	0.506 (0.268-0.954)	.04	1.324 (0.406-4.320)	.64
BCSS	1.252 (0.665-2.358)	.49	0.478 (0.251-0.913)	.03	0.940 (0.435-2.030)	.87	1.336 (0.309-5.774)	.70
Stage								
T1ab								
OS	0.533 (0.290-0.980)	.04	0.446 (0.254-0.782)	.005	1.159 (0.609-2.208)	.65	2.267 (0.980-5.243)	.06
BCSS	1.367 (0.565-3.307)	.49	0.469 (0.191-1.148)	.10	1.454 (0.661-3.200)	.35	2.786 (1.098-7.070)	.03
T1c								
OS	0.564 (0.419-0.760)	<.001	0.812 (0.606-1.088)	.16	0.416 (0.273-0.634)	<.001	2.240 (1.153-4.350)	.02
BCSS	0.821 (0.541-1.245)	.35	0.915 (0.613-1.366)	.66	0.579 (0.338-0.990)	.04	2.916 (1.432-5.938)	.003

Abbreviations: AHR, adjusted hazard ratio; BCS, breast-conserving surgery; BCSS, breast cancer-specific survival; OS, overall survival.

^a^ Other included patients receiving simple mastectomy, radical mastectomy, or other surgical procedures.

For patients with T1a BC, receiving radiotherapy was associated with better OS (AHR, 0.271; 95% CI, 0.081-0.904; *P* = .03) rather than better BCSS. As shown in Table 3, for patients with T1ab BC, receiving chemotherapy (AHR, 0.533; 95% CI, 0.290-0.980; *P* = .04) or radiotherapy (AHR, 0.446; 95% CI, 0.254-0.782; *P* = .005) after BCS both significantly improved OS, whereas no significant BCSS benefit was found. After receiving other surgical procedures, receiving chemotherapy was not associated with survival benefit, and receiving radiotherapy was associated with worse BCSS (AHR, 2.786; 95% CI, 1.098-7.070; *P* = .03). For patients with T1c BC, who were recommended to receive chemotherapy, chemotherapy was associated with improved OS both after BCS (AHR, 0.564; 95% CI, 0.419-0.760; *P* < .001) and other surgery types (AHR, 0.416; 95% CI, 0.273-0.634; *P* < .001). However, receiving chemotherapy was only associated with better BCSS after other surgery types (AHR, 0.579; 95% CI, 0.338-0.990; *P* = .04) rather than BCS. For patients with stage T1c, receiving radiotherapy after BCS was not associated with better OS or BCSS. In addition, patients with stage T1c who received radiotherapy after other surgery types had significantly worse OS (AHR, 2.240; 95% CI, 1.153-4.350; *P* = .02) and BCSS (AHR, 2.916; 95% CI, 1.432-5.938; *P* = .003) than patients with no radiotherapy.

## Discussion

In the present study, adjuvant chemotherapy and radiotherapy were administered to 28.5% (2202) and 16.5% (1278) of the patients with T1 N0 M0 TNBC, respectively, whereas 38.4% (2973) of the patients received both adjuvant therapies (Table 1). Compared with no adjuvant therapy, all adjuvant therapies were associated with improved OS in patients with T1 N0 M0 TNBC. However, only chemotherapy was associated with significantly better BCSS. For patients of all ages, adjuvant chemotherapy after BCS or other surgery both were associated with improved OS but not BCSS. Adjuvant radiotherapy after BCS was associated with better OS and BCSS in patients aged 70 years and older but not in those younger than 70 years. Both adjuvant therapies after BCS were associated with better OS in patients with T1ab BC but not BCSS. For patients with T1c BC, chemotherapy after BCS or other surgery were both associated with improved OS, whereas only chemotherapy after other surgery types was associated with better BCSS. Our results showed that receiving radiotherapy after other surgery was associated with worse survival in most patients with T1 N0 M0 TNBC. Considering adjuvant radiotherapy was not routinely recommended for patients who received simple or radical mastectomy, patients who received radiotherapy after these procedures might have had some clinical risk factors that resulted in a worse prognosis.

Routine chemotherapy is not recommended for patients with T1a TNBC by the consensus guidelines.^10,16^ Our results showed that most patients with T1a BC (43.6%) preferred radiotherapy only, followed by no adjuvant therapy. Further survival analysis revealed that radiotherapy was associated with greater benefit for OS than for BCSS in patients with T1a TNBC. Although the existing evidence is inadequate to support radiotherapy for patients with T1a TNBC, in some clinical practices, these patients would receive routine radiotherapy after BCS. Winzer et al^17^ warned that the absence of radiotherapy after BCS was associated with an increase in the risk of local recurrence. Meanwhile, for patients with TNBC, radiotherapy after BCS or mastectomy could significantly reduce the incidence of BC-caused death and risk of recurrence.^18,19^ Thus, for patients with T1a BC, routine radiotherapy after BCS should be considered, which necessitates further substantiation in a prospective cohort study.

In the present study, 23.2% of the patients with T1b BC received radiotherapy, as many as those who received chemotherapy only (23.3%), whereas most patients with T1b BC received both adjuvant therapies (37.5%). However, further survival analysis revealed no significant survival benefit from adjuvant therapies for patients with T1b BC. The current NCCN guidelines^10^ suggest that chemotherapy should be considered for patients with T1b N0 TNBC, without radiotherapy. Chemotherapy was also recommended by the 15th St. Gallen International Breast Cancer Conference^16^ for patients with stage T1b TNBC or higher. However, in a retrospective study^20^ that included a cohort of 354 patients with T1 N0 M0 TNBC, chemotherapy had no association with better recurrence-free survival in patients with T1b TNBC, whereas it was associated with improved survival in patients with T1c TNBC. The subgroup analysis in another study^21^ reported the absence of significantly better disease-free and metastasis-free survival in patients with T1b TNBC who received adjuvant chemotherapy. Given the limited evidence for survival benefit, the benefit to risk ratio for patients with T1b TNBC should be discussed carefully when adjuvant therapies are considered.

Although various investigational approaches to treatment are emerging, chemotherapy currently remains the primary treatment for TNBC, especially for patients with T1c TNBC.^22^ The efficacies of different chemotherapy regimens have been evaluated in previous studies. Compared with the basal TC6 regimen (docetaxel and cyclophosphamide for 6 cycles), doxorubicin and cyclophosphamide regimens with a taxane improved invasive disease-free survival in patients with TNBC.^23^ Moreover, addition of adjuvant capecitabine to anthracycline- and taxane-based regimens improved the survival outcome and reduced the recurrence of TNBC.^24^ However, the more suitable regimen for early-stage TNBC remains unclear, thus necessitating further study.

Considering the health status of older patients, adjuvant therapies are usually used with caution in these patients, unlike in younger patients.^25^ Half of the older patients were undertreated because of unclear considerations, which consequently led to an adverse prognosis.^26^ The NCCN guidelines^10^ do not recommend adjuvant therapy for older patients owing to limited evidence. According to our results, receiving chemotherapy after surgical procedures was associated with improved OS both of patients younger than 70 years and those 70 years and older, although no significant improvement in BCSS was attained. However, receiving radiotherapy after BCS was associated with better OS and BCSS in patients aged 70 years and older but not in patients younger than 70 years. Thus, clinicians should consider postoperative routine radiotherapy for patients aged 70 years and older who have T1 N0 M0 TNBC based on the evaluation of the patient’s health status, which necessitates further confirmation from a prospective cohort study.

### Strengths and Limitations

The present study has several strengths and limitations. One strength is that it only included patients with T1 N0 M0 TNBC, whereas most previous studies evaluated treatment in cohorts of total BC patients. Another strength is that the present study investigated prognosis in patients with different cancer stages and ages in accordance with the consensus guidelines. Third, despite the low incidence of early-stage TNBC, this study included a large cohort of eligible patients as compared with other studies and evaluated for the first time the association of radiotherapy with OS and BCSS in these patients.

However, this study also has some limitations. First, although we adjusted for multiple confounders, we were unable to evaluate the influence of comorbidities and neoadjuvant therapies on patient prognosis because the required data were unavailable in the SEER database. Second, in the multivariable Cox analysis, loglik converged before the variable of stage when we stratified stage into the T1a, T1b, and T1c subgroups; thus, some results were infinite and not shown in the article. Third, the clinical and therapeutic characteristics in this study were restricted to the United States and might be different in other countries. Lastly, we were unable to address the potential immortal time bias, because date and duration of adjuvant therapies were unavailable in the SEER registry.

## Conclusions

This cohort study found that adjuvant therapies, including chemotherapy, radiotherapy, and both, were associated with improved OS in patients with T1 N0 M0 TNBC, whereas only chemotherapy was associated with better BCSS. Older patients with early-stage TNBC preferred receiving radiotherapy after surgery, which could improve both OS and BCSS as compared with no therapy. Administration of adjuvant therapies to patients with different ages and cancer stages should be discussed carefully, which necessitates guidance from updated guidelines.

## Notes

Supplement. **eTable. **Univariate and Multivariate COX Analysis of Overall Survival in Patients With T1N0M0 TNBC **eFigure. **Flowchart for Included Patients From the SEER
