# Supplementary material for: Evaluation of Adjuvant Treatments for T1 N0 M0 Triple-Negative Breast Cancer
Source: JAMA Netw Open. 2020 Nov 19;3(11):e2021881. doi: 10.1001/jamanetworkopen.2020.21881 (PMC7677762; doi:10.1001/jamanetworkopen.2020.21881)
Supplement: Supplement. — eTable. Univariate and Multivariate COX Analysis of Overall Survival in Patients With T1N0M0 TNBC eFigure. Flowchart for Included Patients From the SEER [file jamanetwopen-e2021881-s001.pdf]

# Supplemental Online Content

Zhai Z, Zheng Y, Yao J, et al. Evaluation of adjuvant treatments for T1 N0 M0 triple-negative breast cancer. *JAMA Netw Open*. 2020;3(11):e2021881. doi:10.1001/jamanetworkopen.2020.21881

**eTable.** Univariate and Multivariate COX Analysis of Overall Survival in Patients With T1N0M0 TNBC

**eFigure.** Flowchart for Included Patients From the SEER

This supplemental material has been provided by the authors to give readers additional information about their work.

© 2020 Zhai Z et al. *JAMA Network Open.*

**eTable.** Univariate and multivariate COX analysis of overall survival in patients with T1N0M0 TNBC.

| Characteristic | Patients.n | Events.n | Rates,% | Univariate Analysis | | Multivariable Analysis | |
|---|---|---|---|---|---|---|---|
| | | | | OS HR (95% CI) | *P* Value | OS AHR (95% CI) | *P* Value |
| Age | | | | | | | |
| 50-70 | 4328 | 194 | 4.48 | Reference | | Reference | |
| <35 | 173 | 10 | 5.78 | 1.297 (0.687-2.448) | 0.42 | 1.199 (0.627-2.290) | 0.58 |
| 35-50 | 1523 | 62 | 4.07 | 0.901 (0.677-1.200) | 0.48 | 0.856 (0.639-1.147) | 0.30 |
| ⩾70 | 1715 | 195 | 11.37 | 2.631 (2.157-3.210) | <0.001 | 2.090 (1.677-2.604) | <0.001 |
| Race | | | | | | | |
| Non-Hispanic White | 4997 | 301 | 6.02 | Reference | | Reference | |
| Non-Hispanic Black | 1377 | 94 | 6.83 | 1.171 (0.929-1.476) | 0.18 | 1.169 (0.923-1.483) | 0.20 |
| Non-Hispanic Other Races | 569 | 28 | 4.92 | 0.864 (0.586-1.272) | 0.46 | 0.910 (0.617-1.342) | 0.63 |
| Hispanic (All Races) | 796 | 38 | 4.77 | 0.877 (0.626-1.229) | 0.45 | 0.924 (0.657-1.300) | 0.65 |
| Marital Status | | | | | | | |
| Married | 4790 | 228 | 4.76 | Reference | | Reference | |
| Single | 1064 | 63 | 5.92 | 1.241 (0.939-1.641) | 0.13 | 1.204 (0.906-1.600) | 0.20 |
| DSW | 1885 | 170 | 9.02 | 1.870 (1.533-2.281) | <0.001 | 1.385 (1.122-1.708) | 0.002 |
| Grade | | | | | | | |
| G1 | 276 | 8 | 2.90 | Reference | | Reference | |
| G2 | 1775 | 105 | 5.92 | 2.162 (1.053-4.436) | 0.04 | 2.254 (1.096-4.637) | 0.03 |
| G3 | 5644 | 340 | 6.02 | 2.242 (1.112-4.519) | 0.02 | 2.653 (1.308-5.379) | 0.007 |
| G4 | 44 | 8 | 18.18 | 6.116 (2.295-16.297) | <0.001 | 6.484 (2.417-17.396) | <0.001 |
| Stage | | | | | | | |
| T1a | 755 | 23 | 3.05 | Reference | | Reference | |
| T1b | 1979 | 85 | 4.30 | 1.329 (0.838-2.106) | 0.23 | 1.549 (0.973-2.466) | 0.06 |
| T1c | 5005 | 353 | 7.05 | 2.287 (1.500-3.488) | <0.001 | 2.829 (1.834-4.363) | <0.001 |
| Surgery | | | | | | | |
| BCS | 5372 | 282 | 5.25 | Reference | | Reference | |
| Simple | 1798 | 133 | 7.40 | 1.456 (1.184-1.789) | <0.001 | 1.325 (1.009-1.739) | 0.04 |
| Radical | 463 | 41 | 8.86 | 1.484 (1.069-2.059) | 0.02 | 1.206 (0.833-1.744) | 0.32 |
| Other | 106 | 5 | 4.72 | 1.328 (0.548-3.218) | 0.53 | 1.855 (0.749-4.593) | 0.18 |
| Adjuvant Therapy | | | | | | | |
| No | 1286 | 146 | 11.35 | Reference | | Reference | |
| Chemotherapy | 2202 | 101 | 4.59 | 0.427 (0.332-0.551) | <0.001 | 0.475 (0.362-0.625) | <0.001 |
| Radiotherapy | 1278 | 88 | 6.89 | 0.580 (0.445-0.756) | <0.001 | 0.726 (0.528-0.997) | 0.04 |
| Both | 2973 | 126 | 4.24 | 0.368 (0.290-0.467) | <0.001 | 0.489 (0.362-0.661) | <0.001 |

**Abbreviations:** TNBC: triple-negative breast cancer; OS: overall survival; HR: hazard ratio; AHR: adjusted hazard ratio; CI: confidence interval; DSW: divorced/separated/widowed; BCS: breast-conserving surgery.

© 2020 Zhai Z et al. *JAMA Network Open.*

**eFigure.** Flowchart for included patients from the SEER.

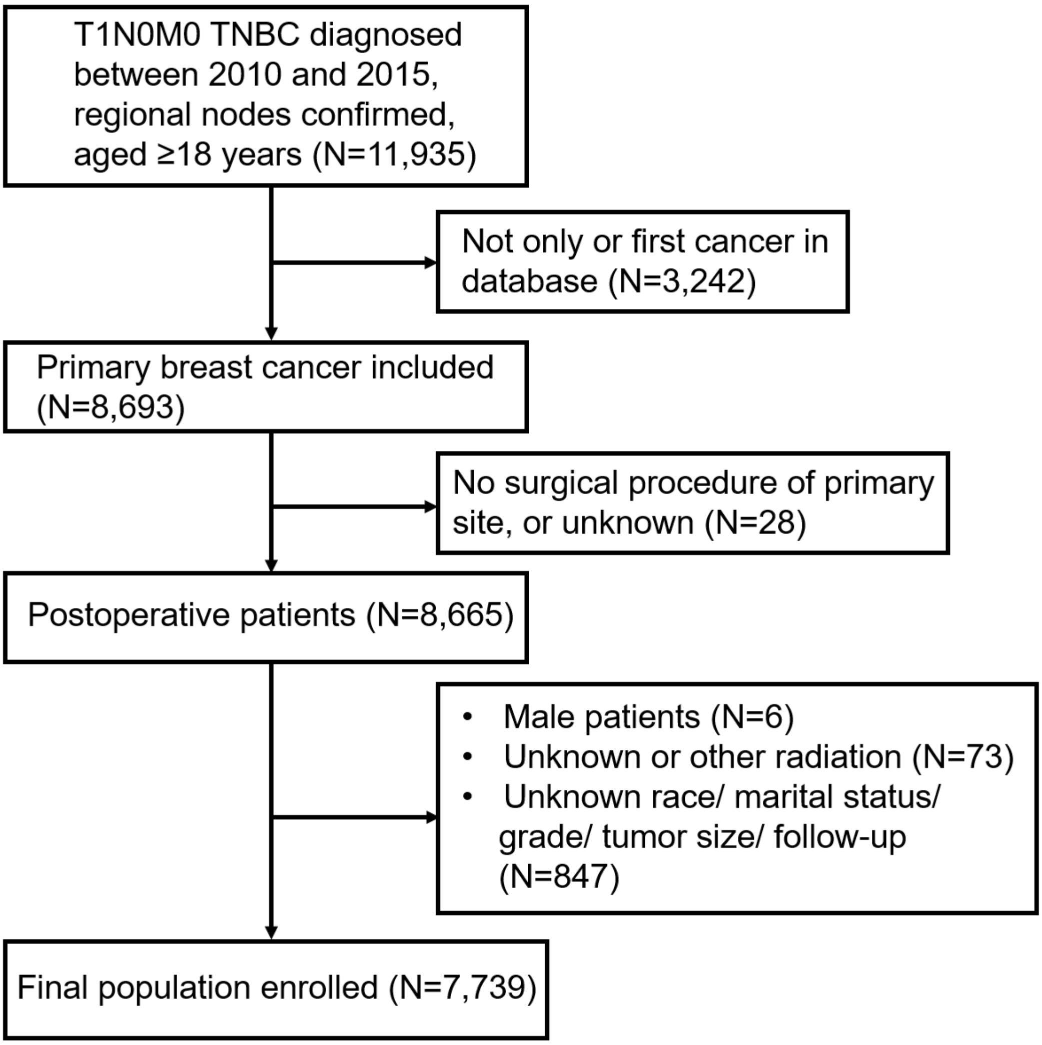


© 2020 Zhai Z et al. *JAMA Network Open.*
